# GTAM: a molecular pretraining model with geometric triangle awareness

**DOI:** 10.1093/bioinformatics/btae524

**Published:** 2024-08-23

**Authors:** Xiaoyang Hou, Tian Zhu, Milong Ren, Bo Duan, Chunming Zhang, Dongbo Bu, Shiwei Sun

**Affiliations:** Key Laboratory of Intelligent Information Processing, Institute of Computing Technology, Beijing, 100049, China; School of Computer Science and Technology, University of Chinese Academy of Sciences, Beijing, 100049, China; Key Laboratory of Intelligent Information Processing, Institute of Computing Technology, Beijing, 100049, China; School of Computer Science and Technology, University of Chinese Academy of Sciences, Beijing, 100049, China; Key Laboratory of Intelligent Information Processing, Institute of Computing Technology, Beijing, 100049, China; School of Computer Science and Technology, University of Chinese Academy of Sciences, Beijing, 100049, China; Key Laboratory of Intelligent Information Processing, Institute of Computing Technology, Beijing, 100049, China; Western Institute of Computing Technology, Chongqing, 400000, China; Key Laboratory of Intelligent Information Processing, Institute of Computing Technology, Beijing, 100049, China; Western Institute of Computing Technology, Chongqing, 400000, China; Phil Rivers Technology, Beijing, 100190, China; Key Laboratory of Intelligent Information Processing, Institute of Computing Technology, Beijing, 100049, China; School of Computer Science and Technology, University of Chinese Academy of Sciences, Beijing, 100049, China; Central China Artificial Intelligence Research Institute, Henan Academy of Sciences, Zhengzhou, 450046, China; Key Laboratory of Intelligent Information Processing, Institute of Computing Technology, Beijing, 100049, China; School of Computer Science and Technology, University of Chinese Academy of Sciences, Beijing, 100049, China; Western Institute of Computing Technology, Chongqing, 400000, China

## Abstract

**Motivation:**

Molecular representation learning is pivotal for advancing deep learning applications in quantum chemistry and drug discovery. Existing methods for molecular representation learning often fall short of fully capturing the intricate interactions within chemical bonds of 2D topological graphs and the multifaceted effects of 3D geometric conformations.

**Results:**

To overcome these challenges, we present a novel contrastive learning strategy for molecular representation learning, named **G**eometric **T**riangle **A**wareness **M**odel (GTAM). This method integrates innovative molecular encoders for both 2D graphs and 3D conformations, enabling the accurate capture of geometric dependencies among edges in graph-based molecular structures. Furthermore, GTAM is bolstered by the development of two contrastive training objectives designed to facilitate the direct transfer of edge information between 2D topological graphs and 3D geometric conformations, enhancing the functionality of the molecular encoders. Through extensive evaluations on a range of 2D and 3D downstream tasks, our model has demonstrated superior performance over existing approaches.

**Availability and implementation:**

The test code and data of GTAM are available online at https://github.com/StellaHxy/GTAM.

## 1 Introduction 

Deep learning has increasingly influenced molecular chemistry, yielding notable progress in quantum chemistry (Dral 2020) and drug discovery (Stokes et al. 2020, Liu et al. 2023b). Acquiring molecular representation embeddings is crucial for advancing deep learning in this field. These embeddings are key in the design of functional and innovative compounds using deep learning methodologies (Jumper et al. 2021).

Molecules can be depicted through two distinct modalities: two-dimensional (2D) graphs and three-dimensional (3D) conformations. The 2D graphs highlight the topological connections among atoms, represented by nodes (atoms) and edges (chemical bonds). The 3D conformations focus on spatial atom arrangements, defined by each atom’s specific spatial coordinates within the molecule. Recent studies (Schütt et al. 2017, Luo et al. 2022, Stärk et al. 2022, Zhou et al. 2022, Liu et al. 2023a) have demonstrated that enhancing the mutual information between these two modalities improves molecular representation capabilities. Notable multimodal pretraining approaches for molecules, such as GraphMVP (Liu et al. 2021) and 3DInfomax (Stärk et al. 2022), utilize various objectives to facilitate the exchange of multimodal information. These methods rely fundamentally on the encoders for 2D graphs and 3D conformations.

Existing encoders used for molecular embedding exhibit certain limitations (Garg et al. 2020). First, conventional 2D graph neural networks like the GIN (Xu et al. 2018) and Graphormer (Ying et al. 2021) update node embeddings via edge information aggregation and vice versa. In chemistry, a chemical bond is often influenced by other chemical bonds to which it is connected (Ruedenberg 1962) which highlights the intricate interdependencies within a molecule’s structure. Nevertheless, current graph neural networks used to learn molecular representation embeddings do not adequately model the interrelations of chemical bonds in molecules. Second, for 3D conformation, the inter-edge relationships, which involve physical constraints, have not been effectively addressed in previous graph networks, such as SchNet (Schütt et al. 2017) and DimeNet (Gasteiger et al. 2020b). GeomGCL (Li et al. 2022) attempted to address this issue by proposing edge-to-edge updates in graph data, yet this approach only considers the angle relation between edges. Moreover, most current multimodal pretraining approaches (Liu et al. 2021, Stärk et al. 2022, Zhu et al. 2022, Liu et al. 2023a) focus on maximizing mutual information (MI) across modalities, thereby ensuring that learned representations capture molecular shared information. These methods all focus on the exchange of node information in molecules. Notably, there is a correlation between edge attribute information in 2D topological graphs and edge length information in 3D geometric conformations of molecules (Ruedenberg 1962). The types of chemical bonds significantly influence molecular physical and chemical properties, such as boiling points and solubility. Understanding these bond types and the corresponding distances between atoms is crucial for comprehending and predicting molecular property (Pauling 1931). Yet, previous methods have limitations in effectively learning and sharing edge-related information across different modalities during pretraining.

To address these limitations, we introduce a molecular contrastive learning method called **G**eometric **T**riangle **A**wareness **M**odel (GTAM). GTAM aims to maximize the mutual information using contrastive self-supervised learning (SSL) and generative SSL (Liu et al. 2021, 2023a). First, we use diffusion generative models for generative SSL which can lead to a more accurate estimation in generative SSL. Second, to enhance molecular representations in contrastive SSL, we introduce new molecular encoders that incorporate a novel geometric triangle awareness mechanism to enhance edge-to-edge updates in molecular representation learning, in addition to node-to-edge and edge-to-node updates, unlike other molecular graph encoders (Schütt et al. 2017). Drawing inspiration from the triangle update methods utilized in AlphaFold2 (Jumper et al. 2021), our geometric triangle awareness update mechanism employs a self-attention mechanism for the dynamic integration of other edges and structural information. Third, to reduce information loss in contrastive SSL, GTAM also incorporates two contrastive training objectives to enhance the fusion of edge information across the different modalities. With advanced molecular encoders and our contrastive training objectives, GTAM has demonstrated state-of-the-art performance in downstream tasks both for 2D graphs and 3D conformations, our model works best on 22 out of 28 tasks compared with previous methods.

## 2 Materials and methods

### 2.1 Preliminaries

We employ $G^{\left\{2D,3D\right\}}=(V^{\left\{2D,3D\right\}},E^{\left\{2D,3D\right\}})$ to represent the 2D topological graph and 3D geometric conformation of a molecule. In the 2D topological graph, atoms and chemical bonds are respectively represented as nodes $u\in V^{2D}$ and edges $e\in E^{2D}$. In the 3D geometric conformation, each atom is denoted as a node in the set $V^{3D}$, and we construct a fully connected graph with the distance map comprising the edges $E^{3D}$. More details of molecular featurization are exhibited in Supplementary Appendix A.1.

### 2.2 Contrastive framework

**Figure 1.**
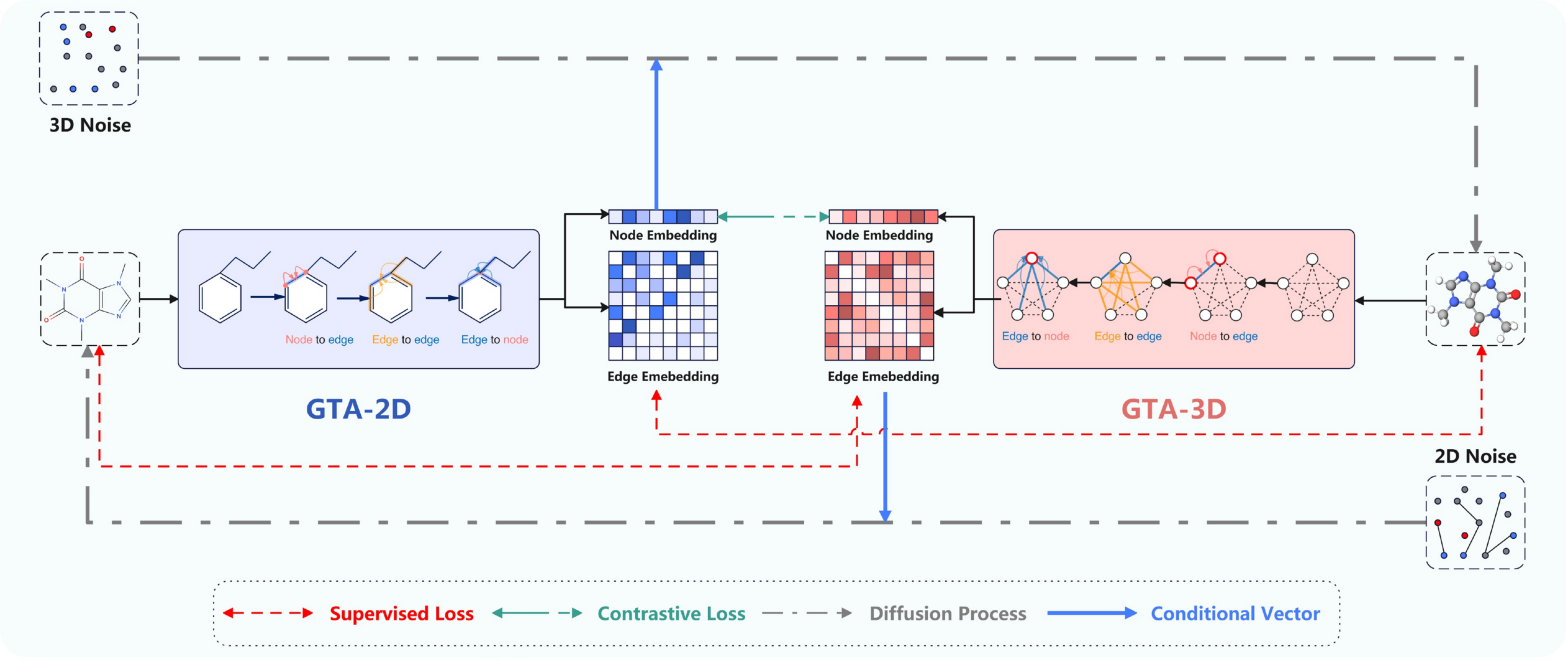
Overview of GTAM: The GTAM framework integrates a geometric triangle awareness updating mechanism along with innovative training objectives. This approach is designed to enhance molecular representation through a comprehensive understanding of the geometric interrelationships within molecular structures

To obtain powerful molecular representation from 2D graphs and 3D conformations, GTAM adopts a contrastive learning strategy to maximize the mutual information between 2D graphs and 3D conformations like previous work (Stärk et al. 2022, Liu et al. 2023a) (Fig. 1) and aims to maximize the conditional generative probability for 3D molecular representations given their corresponding 2D representations, and vice versa. The maximization of mutual information (MI) is reformulated as the summation of two conditional log-likelihoods.

(1) $$\begin{array}{c} L_{\text{MI}}=\frac{1}{2}[-H(C|T)-H(T|C)]\\ =\frac{1}{2}E_{p(T,C)}[\text{log}\;p(C|T)+\text{log}\;p(T|C)]. \end{array}$$

Here, we use **T** and **C** for the 2D and 3D graphs for notation simplicity, i.e. $T≜G^{2D}$ and $C≜G^{3D}$. GTAM leverages a diffusion generative model approach to approximate these conditional probabilities, along with two novel molecular encoders for 2D graphs and 3D conformations. We introduce an additional mechanism for maximizing MI, named GTAInfomax. The training objective of GTAInfomax aims to maximize the paired 2D and 3D molecular representations and minimize the similarity between unpaired representations within the same batch. The details and comprehensive explanation of GTAInfomax are thoroughly documented in Supplementary Appendix A.2.

### 2.3 Molecular encoder

Our approach incorporates the geometric triangle awareness update mechanism within both the 2D graph encoders (GTA-2D) and 3D conformation encoders (GTA-3D). This module includes three updating components: node-to-edge, edge-to-edge, and edge-to-node, which capture comprehensive interrelated information among the elements of a molecular graph. The following sections will be divided into two parts: First, we describe the detailed implementation of these components, and then we elaborate on our molecular graph encoders for 2D graphs and 3D conformations.

#### 2.3.1 Node to edge

First, we employ node embedding *h_u_* to update edge embedding *z_uv_*, with the updating process illustrated as follows:

(2) $$\begin{array}{c} z_{uv}=\phi (z_{uv},f(\{h_{u},h_{v}\})),\\ f(h_{u})=\text{Linear}(\text{LayerNorm}(h_{u})),\\ \phi (z_{uv},f(\{h_{u},h_{v}\}))=z_{uv}+\text{Linear}(f(h_{u})⊗f(h_{v})). \end{array}$$

#### 2.3.2. Edge to edge

To capture the intricate interdependence between edges within the molecular graph, we use two different triangle updates.

(3) $$\begin{array}{l} z_{uv}=\\ \phi_{\left\{1,2\right\}}(z_{uv},f_{\left\{1,2\right\}}(\{z_{uw},z_{vw}\}\;|\;w\in N(u)\cup N(v))). \end{array}$$

The edge update process mainly comes in two forms. The *first* is to directly update the third edge using the information of the two adjacent edges in a triangle. The *second* method involves updating through an attention mechanism.

In the *first* updating forms, we update the edge embedding *z_uv_* through a linear layer.

(4) $$f_{1}(z_{uv})=\text{LayerNorm}(z_{uv}),$$

(5) $$\begin{array}{l} \phi_{1}(z_{uv},f_{1}(\{z_{uw},z_{vw}\}))=\\ \sigma (z_{uv})⊙\text{Linear}(\text{LayerNorm}(\sum_{w\in N(u)\cup N(v)}f_{1}(z_{uw})⊙f_{1}(z_{vw}))). \end{array}$$

In the *second* updating forms, the function *f*_2_ is utilized to project the edge information *z_uv_* through different linear layers into the form of an attention mechanism and a bias. *z_uv_* through different linear layers to obtain the $q_{uv}^{e},k_{uv}^{e},v_{uv}^{e}$ in the attention mechanism and the bias *b_uv_* in the triangle update.

(6) $$\begin{array}{r} q_{uv}^{e},k_{uv}^{e},v_{uv}^{e}\leftarrow \text{Linear}(z_{uv}),\\ b_{uv}\leftarrow \text{Linear}(z_{uv}). \end{array}$$

Subsequently, the function $\phi_{2}$ is updated through an attention-based approach. Differently, in the computation of the attention scores, the information of the third edge is incorporated as a bias term.

(7) $$\begin{array}{cc} a_{\mathrm{uvw}} & =\frac{\;\text{exp}(\frac{1}{\sqrt{c}}q_{uv}^{e}k_{wv}^{e}+b_{wu}^{e})}{\sum_{w\in N(u)\cup N(v)}\;\text{exp}\;(\frac{1}{\sqrt{c}}q_{uv}^{e}k_{wv}^{e}+b_{wu}^{e})},\\ z_{uv} & =z_{uv}+\sigma (z_{uv})⊙(\sum_{w\in N(u)\cup N(v)}a_{\mathrm{uvw}}v_{uw}^{e}). \end{array}$$

#### 2.3.3 Edge to node

To update the information of nodes based on the distance or chemical bond information on the edges, we subsequently use the method of edge-to-node updates.

(8) $$\begin{array}{c} h_{u}=\phi_{\left\{1,2\right\}}(h_{u},f_{\left\{1,2\right\}}(\{z_{uv}\})|u,v\in N(u)). \end{array}$$

We aggregate the node information and edge information separately along rows and columns.

(9) $$\begin{array}{c} f_{\left\{1,2\right\}}(z_{uv})=\text{LayerNorm}(z_{uv}),\\ \phi_{1}(h_{u},f_{1}(\{z_{uv}\}))=h_{u}+\text{MLP}(\sum_{u\in N(v)}f(z_{uv})),\\ \phi_{2}(h_{u},f_{2}(\{z_{uv}\}))=h_{u}+\text{MLP}(\sum_{v\in N(u)}f(z_{uv})). \end{array}$$

After that, we update the obtained normalized node information through a three-layer multi-layer perceptron. For a thorough understanding of the computational efficiency and scalability of the geometric triangle awareness update mechanism, an in-depth analysis of the time complexity has been conducted in Supplementary Appendix A.3.

To enhance molecular encoding through the integration of geometric triangle awareness updates, we employ a tailored approach for both 2D and 3D molecular structures. In 2D graphs, GTA-2D initially processes nodes and edges using two layers of the graph isomorphism network (Xu et al. 2018). These initial layers enable us to obtain fundamental embeddings for nodes and edges, capturing two-hop information within the graphs. We then apply our geometric triangle awareness update method in the 2D graphs. This method is adept at efficiently capturing global edge information and updating edge embeddings with other edges. The node and edge embeddings are denoted as $h_{u}^{2D}$ and $z_{uv}^{2D}$.

Regarding 3D conformations, GTA-3D first encodes atom coordinates and nuclear charges with a layer of cfconv (Schütt et al. 2017) to establish basic embeddings. Following this, we implement the geometric triangle awareness update within a fully connected graph for 3D conformations. Owing to the nature of the fully connected structure, our update method can rapidly capture all atom–atom interactions and incorporate these many-body effects into both node and edge embeddings, which are denoted as $h_{u}^{3D}$ and $z_{uv}^{3D}$.

### 2.4 Diffusion processes

To effectively estimate the conditional probabilities log p(C|T) and log p(T|C), we utilize diffusion generative models which are adept at capturing the complex transition from 2D graphs, denoted as $T=G^{2D}$, to 3D conformations, represented by $C=G^{3D}$, and vice versa.

For the 2D graph **T** to 3D conformation **C** generation task, the forward SDE can be denoted as:

(10) $$dx_{t}=f_{1}(x_{t})dt+g_{1}(t)dw_{t}.$$

For the 3D conformation **C** to 2D graph **T** generation task, the forward SDE can be formulated as:

(11) $$\{\begin{array}{c} dV_{t}^{2D}=f_{2}(V_{t}^{3D},E_{t}^{2D})dt+g_{2}(t)dw_{t}^{1}\\ dE_{t}^{2D}=f_{3}(V_{t}^{2D},E_{t}^{2D})dt+g_{3}(t)dw_{t}^{2} \end{array},$$

where $f_{1},f_{2},f_{3}$ and $g_{1},g_{2},g_{3}$ denote the drift and diffusion coefficients and $w_{t},w_{t}^{1},w_{t}^{2}$ represents independent Brownian motions, and we set these hyperparameters and score networks for diffusion process same as previous work (Liu et al. 2023a).

We utilize the GTA-2D and GTA-3D as our conditional encoder to encode 2D graphs and 3D conformations.

$$h^{2D},z^{2D}\leftarrow \text{GTA}-2D(T),\;h^{3D},z^{3D}\leftarrow \text{GTA}-3D(C).$$

For the 2D graphs $G^{2D}$ to 3D conformations $G^{3D}$ generation task, the score network $S_{\theta }^{2D\to 3D}(C_{t},h^{2D},t)$ is SE(3)-equivariant score network, and the score network $S_{\theta }^{3D\to 2D}(T_{t},h^{3D},t)$ is SE(3)-invariant score network for the 3D conformations $G_{3D}$ to 2D graphs $G_{2D}$ generation task.

### 2.5 Training objectives

To maximize the MI $L_{\text{MI}}$, GTAM has several pretraining tasks: (i) contrastive learning between 2D and 3D molecular representations; (ii) generation of 3D conformations based on 2D graphs, to create 3D molecular structures from 2D graphical inputs; (iii) generation of 2D graphs conditional upon 3D conformations, emphasizing the derivation of 2D topological graphs from 3D geometric conformations.

First, the contrastive objective can be formulated as:

(12) $$\begin{array}{l} L_{\text{Contrastive}}=\eta_{1}L_{\text{node}}+\eta_{2}L_{\text{edge}},\\ L_{\text{node}}=L_{2\text{Dnode}}+L_{3\text{Dnode}}\\ L_{2\text{Dnode}}=-\frac{1}{2}E_{p(C)}[E_{p_{t}(T_{t}|T,C)}\;\text{log}(1-\sigma (S_{\theta }^{3D\to 2D}(T_{t},h^{3D},t)))\\ +E_{p(C|T)}\;\text{log}\;\sigma (S_{\theta }^{3D\to 2D}(T_{t},h^{3D},t))]\\ L_{3\text{Dnode}}=-\frac{1}{2}E_{p(T)}[E_{p_{t}(C_{t}|C,T)}\;\text{log}(1-\sigma (S_{\theta }^{2D\to 3D}(C_{t},h^{2D},t)))\\ +E_{p(C|T)}\;\text{log}\;\sigma (S_{\theta }^{2D\to 3D}(C_{t},h^{2D},t))], \end{array}$$

where $p_{t}(T_{t}|T,C)$ and $p_{t}(C_{t}|C,T)$ are the noise distribution during diffusion process and *σ* is the sigmoid function.

Expanding upon the foundational concept of contrastive learning of node embeddings, we incorporate two innovative contrastive training objectives specifically designed to enhance the transfer and integration of edge information across 2D graphs and 3D conformations. These objectives, the contrastive loss for 3D edge $L_{3\text{Dedge}}$ and the contrastive loss for 2D edge $L_{2\text{Dedge}}$, collectively form the contrastive objective for edge information, defined as:

(13) $$L_{\text{edge}}=L_{2\text{Dedge}}+L_{3\text{Dedge}}.$$

These objectives are designed to enhance the fidelity and relevance of edge embeddings across different molecular representations by implementing direct edge constraints.

For the contrastive loss for the edges in 2D graphs, we employ the 3D conformation distance map as a label and project the edge embeddings from the 2D graphs to predict this distance map. We discretize the distance between atoms *u* and *v* in 3D conformations into 32 bins ranging from 1.0 Å to 2.4 Å, with this specific range being determined based on estimates derived from our pretraining dataset, encoding them into one-hot vectors $d_{uv}^{3D}$. The edge embeddings are then projected into these 32 distance bins to obtain bin probabilities $p_{uv}^{2D}$ using softmax. The loss is calculated as follows:

(14) $$L_{2\text{Dedge}}=-\frac{1}{\left|E_{2D}\right|}\sum_{u,v\in V_{2D}}\sum_{b=1}^{32}d_{uv}^{3D}\;\text{log}\;p_{uv}^{2D}.$$

For the contrastive loss for the edges in 3D conformations, the chemical bond attributes from 2D graphs are used as labels $y_{uv}^{2D}$, with an additional label for the absence of a chemical bond. We project the pair embeddings $z_{uv}^{3D}$ from 3D conformations to predict these labels, employing a cross-entropy loss for the classification task:

(15) $$L_{3\text{Dedge}}=-\sum_{e_{uv}\in E}y_{uv}^{2D}\;\text{log}\;z_{uv}^{3D}.$$

The second objective is the conditional generation from 2D graphs to 3D conformations. The goal is to use $S_{\theta }^{2D\to 3D}$ to estimate the score $\nabla_{C_{t}}\;\text{log}\;p_{t}(C_{t}|C,T)$. To learn p(C|T), based on the score network, the training objective is:

(16) $$\begin{array}{c} L^{2D\to 3D}=\\ E_{T,C}E_{t}E_{C_{t}|C}[||\nabla_{C_{t}}\;\text{log}\;p_{t}(C_{t}|C,T)-S_{\theta }^{2D\to 3D}(C_{t},h^{2D},t){|}_{2}^{2}]. \end{array}$$

The third objective is reconstructing the 2D graphs from 3D conformations, p(x|y). The goal is to use $S_{\theta }^{3D\to 2D}$ to estimate the score $\nabla_{T_{t}}\;\text{log}\;p_{t}(T_{t}|T,C)$. The training objective for learning p(T|C) can be formulated as:

(17) $$\begin{array}{c} L^{3D\to 2D}=\\ E_{T,C}E_{t}E_{T_{t}|T}[||\nabla_{T_{t}}\;\text{log}\;p_{t}(T_{t}|T,C)-S_{\theta }^{3D\to 2D}(T_{t},h^{3D},t){|}_{2}^{2}]. \end{array}$$

The comprehensive training objective for the GTAM encapsulates a multifaceted approach to molecular representation learning, and the overall training objective is articulated as follows:

(18) $$L=\alpha_{1}L_{\text{Contrastive}}+\alpha_{2}L^{2D\to 3D}+\alpha_{3}L^{3D\to 2D}$$

## 3 Experiments

We pre-trained our model on PCQM4Mv2, where molecules are represented by both 2D graphs and corresponding 3D conformations. We evaluated 28 distinct tasks to ascertain the effectiveness of our method, categorized as follows: (i) eight molecular property tasks utilizing solely 2D topological graphs in the MoleculeNet dataset; (ii) 20 regression tasks based on 3D geometric conformations in the QM9 and MD17 datasets. We also use molecular retrieval tasks to validate our model’s practical application. Finally, we conduct extensive ablation studies to investigate the impact of geometric triangle awareness update methods. A detailed description of the experiment and the datasets can be found in Supplementary Appendices A and B.

### 3.1 Dataset

PCQM4Mv2 dataset has 3.38M molecules in total. It is a quantum chemistry dataset originally curated under the PubChemQC (Nakata and Shimazaki 2017) project with both 2D topological graphs and 3D geometric conformations.

MoleculeNet (Wu et al. 2018) is a large-scale benchmark for molecular machine learning, which curates multiple public datasets and establishes metrics for evaluation. We choose eight binary classification tasks on MoleculeNet: BACE, BBBP, ClinTox, HIV, SIDER, Tox21, ToxCast, and MUV. Most of them are with limited data. We choose the Toxcast dataset in the MoleculeNet dataset to conduct molecular retrieval experiments.

MD17 (Chmiela et al. 2017) is a dataset for molecular dynamics simulations. It comprises eight different organic molecules, each corresponding to a specific task. It aims to predict the energy-preserving forces for each atom in the molecule. QM9 (Ramakrishnan et al. 2014) is a subset of the GDB-17 database and comprises 134 thousand stable organic molecules consisting of nine heavy atoms. It has 12 tasks related to the quantum properties.

### 3.2 Baselines

For 2D graph tasks, our approach is compared with a range of 2D pretraining models such as Deep Graph Infomax (Veličković et al. 2018), AttrMask (Liu et al. 2019), ContextPred (Hu et al. 2019), GraphCL (You et al. 2020), InfoGraph (Sun et al. 2022), Mole-BERT (Xia et al. 2022), and MoleMCL (Zhang et al. 2024). We also compare with three 2D–3D multimodality pretraining baselines: GraphMVP (Liu et al. 2021), 3DInfomax (Stärk et al. 2022), and MoleculeSDE (Liu et al. 2023a).

For 3D conformation tasks, we follow the baselines from GeoSSL (Liu et al. 2022). The study introduces four coordinate-MI-unaware SSL baselines, two contrastive SSL baselines, and one generative SSL baseline, predicting three key molecular aspects: identifying hidden atom types, estimating distances between atom pairs, and calculating angles among atom triplets. 3D InfoGraph discerns whether node and graph-level 3D representations correspond to the same molecule. We utilize three distinct objective functions, including RR, InfoNCE, and EBM-NCE, to maximize the MI between the conformations and its augmented counterpart. We also evaluate our method by comparing it with several notable models including GraphMVP, 3D InfoMax, Zaidi et al. (Zaidi et al. 2022), GeoSSL, and MoleculeSDE.

For these datasets, we established no pretraining baselines, building models with random weight initialization without pretraining, to comparatively assess the effectiveness of pretraining strategies by evaluating model performance pre- and post-pretraining.

### 3.3 Results on 2D graph tasks

For each task, we split the dataset into training, validation, and test sets by 8:1:1 according to molecular scaffolds according to the scaffold split. The results of the classification tasks on MoleculeNet are summarized in Table 1. We can observe that our work demonstrates superior performance in molecular property prediction when compared to previous methods.

**Table 1.** Results on MoleculeNet dataset with 2D topological graphs only. For each downstream task, we present the mean ROC-AUC (with standard deviation) across three seeds, using scaffold splitting. The best and second-best results are marked in bold and underlined, respectively.

Dataset	BACE	BBBP	ClinTox	HIV	Sider	Tox21	ToxCast	MUV	Avg
no pretraining	80.95 ± 0.92	69.85 ± 0.86	82.83 ± 0.71	74.28 ± 1.32	58.87 ± 1.21	76.55 ± 0.31	63.93 ± 0.19	78.60 ± 2.02	73.16 ± 0.94
AttrMask	78.29 ± 0.47	67.90 ± 2.21	86.49 ± 1.27	75.94 ± 0.61	60.51 ± 0.14	74.38 ± 0.26	62.93 ± 0.13	73.70 ± 1.97	72.50 ± 0.88
GraphCL	75.62 ± 0.93	69.09 ± 1.03	71.33 ± 1.28	76.39 ± 2.04	60.12 ± 0.91	74.01 ± 0.42	62.87 ± 0.35	75.27 ± 1.69	70.59 ± 1.08
ContextPred	79.97 ± 2.28	65.09 ± 0.64	75.63 ± 0.78	75.07 ± 0.93	62.03 ± 0.56	74.02 ± 0.12	61.91 ± 0.41	74.73 ± 1.51	71.06 ± 0.90
InfoGraph	78.52 ± 0.93	66.53 ± 0.63	74.81 ± 1.11	74.97 ± 1.03	58.91 ± 0.20	73.49 ± 0.52	63.95 ± 0.63	73.97 ± 0.85	70.64 ± 0.73
GraphMVP	80.43 ± 0.79	68.54 ± 0.32	84.07 ± 1.82	75.62 ± 0.79	59.42 ± 0.23	75.93 ± 0.48	65.83 ± 0.19	75.01 ± 2.14	73.11 ± 0.84
3DInfomax	80.66 ± 1.46	68.39 ± 0.21	80.54 ± 2.97	75.54 ± 0.65	59.23 ± 0.68	73.31 ± 0.15	65.39 ± 0.29	75.32 ± 2.33	72.30 ± 1.09
Mole-BERT	80.34 ± 0.99	69.91 ± 0.46	82.07 ± 1.61	76.07 ± 1.26	60.38 ± 0.47	76.15 ± 0.27	66.02 ± 0.32	76.26 ± 2.07	73.40 ± 0.93
MoleculeSDE	81.05 ± 1.17	69.21 ± 0.35	84.53 ± 3.01	76.13 ± 0.37	59.69 ± 0.72	76.41 ± 0.37	65.27 ± 0.30	79.08 ± 0.27	73.96 ± 0.82
MoleMCL	81.04 ± 0.79	68.50 ± 1.71	85.79 ± 1.79	77.37 ± 0.59	59.47 ± 0.77	76.19 ± 0.63	66.11 ± 0.58	78.67 ± 1.29	74.14 ± 1.02
GTAInfomax	82.06 ± 0.56	70.34 ± 0.37	88.28 ± 0.92	**78.48 ± 0.77**	61.08 ± 0.59	74.30 ± 0.54	66.14 ± 0.29	78.35 ± 0.81	74.88 ± 0.61
GTAM	**85.23 ± 0.16**	**70.65 ± 0.49**	**88.37 ± 0.53**	77.52 ± 0.22	**63.89 ± 0.62**	**77.59 ± 0.36**	**66.48 ± 0.14**	**79.51 ± 0.47**	**76.13 ± 0.37**

To validate the effectiveness of our molecular encoders, the results of GTAInfomax are also presented in Table 1 and have achieved impressive outcomes that are better than some previous works such as 3DInfomax. Additionally, according to Table 1, some pretraining baselines such as ContextPred and AttrMask perform similarly or even worse than our no pretraining baseline, which can demonstrate the superior performance of our molecular encoder in supervised learning. Moreover, GTAM’s performance on these datasets exceeded that of our no pretraining baseline, further illustrating the effectiveness of the pretraining phase in enhancing performance in downstream tasks. These indicate the effectiveness of our method in extracting molecular feature information and integrating information between 2D graphs and 3D conformations modalities.

### 3.4 Results on 3D conformation tasks

In the MD17 dataset, following previous works (Schütt et al. 2017, Gasteiger et al. 2020a), we use 1K molecules for finetuning, 1K for validation, and 48–991K for testing across a variety of tasks. The goal of the MD17 is to predict the energy-conserving interatomic forces for each atom at each molecule position. We can observe that GTAM works best on seven out of eight in Table 2. For the QM9 dataset, we take 110 thousand molecules for the training set, 10 thousand for validation, and 11 thousand for the testing segment. The results, displayed in Table 3, can reach the best performance on 7 tasks.

**Table 2.** Results on eight atomic forces predictions on the MD17 dataset (in kcal/mol/Å) with 3D geometric conformations only. The evaluation is mean absolute error. The best and second-best results are marked in bold and underlined, respectively.

Dataset	Aspirin	Benzene	Ethanol	Malonaldehyde	Naphthalene	Salicylic	Toluene	Uracil
no pretraining	1.0631	0.3547	0.3770	0.8762	0.6005	0.7775	0.5125	0.5739
Type Prediction	1.2497	0.3942	0.4374	0.8661	0.6224	1.0199	0.6691	0.8590
Distance Prediction	1.3425	0.3987	0.4139	0.8592	0.7610	0.8914	0.5104	1.6628
Angle Prediction	1.3847	0.4485	0.6877	1.1060	0.6715	1.0472	0.6359	0.7706
3D InfoGraph	1.4439	0.4291	0.5404	0.8924	0.7798	1.2378	0.7621	1.1403
RR	1.1014	0.3853	0.5159	1.0876	0.5970	0.8419	0.5616	0.7193
InfoNCE	1.1149	0.3904	0.4748	0.8872	0.5351	0.8284	0.5578	0.6606
EBM-NCE	1.0871	0.3788	0.4574	0.8202	0.5133	1.0067	0.5602	0.7528
GraphMVP	1.1028	0.3701	0.4432	0.7192	0.4819	0.7379	0.5003	0.6094
Zaidi et al.	1.2235	0.3987	0.4374	0.8202	0.5873	0.8046	0.6190	0.5917
GeoSSL	1.0193	0.3853	0.3433	0.7352	0.5758	0.8943	0.4879	0.5065
MoleculeSDE	1.0528	0.2961	0.2979	0.5103	0.4363	0.7115	0.4968	**0.4504**
GTAM	**0.8437**	**0.2658**	**0.2822**	**0.5008**	**0.3442**	**0.6981**	**0.4063**	0.4552

**Table 3.** Results on 12 energy predictions on the QM9 dataset using 110K for training with 3D geometric conformations only. The evaluation is mean absolute error. The best and second-best results are marked in bold and underlined, respectively.

Dataset	Alpha	Gap	HOMO	LUMO	Mu	Cv	G298	H298	R2	U298	U0	Zpve
no pretraining	0.060	44.01	27.67	22.04	0.0274	0.031	14.07	12.15	0.134	13.77	13.41	1.812
Type Prediction	0.074	44.35	28.93	22.49	0.0351	0.033	17.12	16.34	0.283	15.73	14.79	2.143
Distance Prediction	0.064	46.02	27.54	23.78	0.0323	0.035	15.06	15.73	0.251	15.32	15.18	1.876
Angle Prediction	0.066	47.94	28.96	24.38	0.0328	0.031	14.25	13.86	0.223	13.47	13.55	1.859
3D InfoGraph	0.061	46.05	29.54	24.46	0.0283	0.030	14.04	14.02	0.138	13.42	13.54	1.638
RR	0.060	43.19	28.12	22.36	0.0296	0.030	14.47	13.67	0.121	13.99	13.82	1.706
InfoNCE	0.060	44.81	28.26	22.93	0.0273	0.030	13.27	13.42	**0.119**	13.02	13.04	1.658
EBM-NCE	0.058	43.69	27.09	22.62	0.0295	0.030	12.91	12.54	0.127	13.24	12.71	1.659
GraphMVP	0.057	42.16	25.86	21.57	0.0275	0.029	13.48	13.37	0.139	12.97	13.02	1.612
3DInfomax	0.057	42.67	25.86	21.58	0.0288	0.030	13.64	13.59	0.142	13.78	13.28	1.669
Zaidi et al.	0.058	43.17	26.17	22.02	0.0281	0.030	12.63	12.76	0.171	12.43	12.14	1.687
GeoSSL	0.057	42.37	**25.73**	22.09	0.0274	0.029	**11.56**	11.16	0.169	11.10	11.04	1.658
MoleculeSDE	0.056	41.65	25.85	21.64	0.0268	0.028	12.71	**11.03**	0.203	11.08	11.07	**1.507**
GTAInfomax	0.057	41.52	25.86	**21.47**	0.0266	0.029	13.07	11.67	0.138	12.33	12.13	1.604
GTAM	**0.056**	**41.44**	25.84	21.57	**0.0252**	**0.028**	12.62	11.11	0.137	**11.08**	**10.96**	1.558

We also tested the GTAInfomax on the QM9 dataset and MD17 dataset, and it demonstrates superior performance compared to other models. Moreover, compared to our no pretraining baseline, some pretraining baselines show similar or poorer results on the 20 3D conformation tasks. These results highlight the efficacy of our GTA-3D. More results about GTAM can be found in Supplementary Appendix C.

### 3.5 Results on molecular retrieval

**Figure 2.**
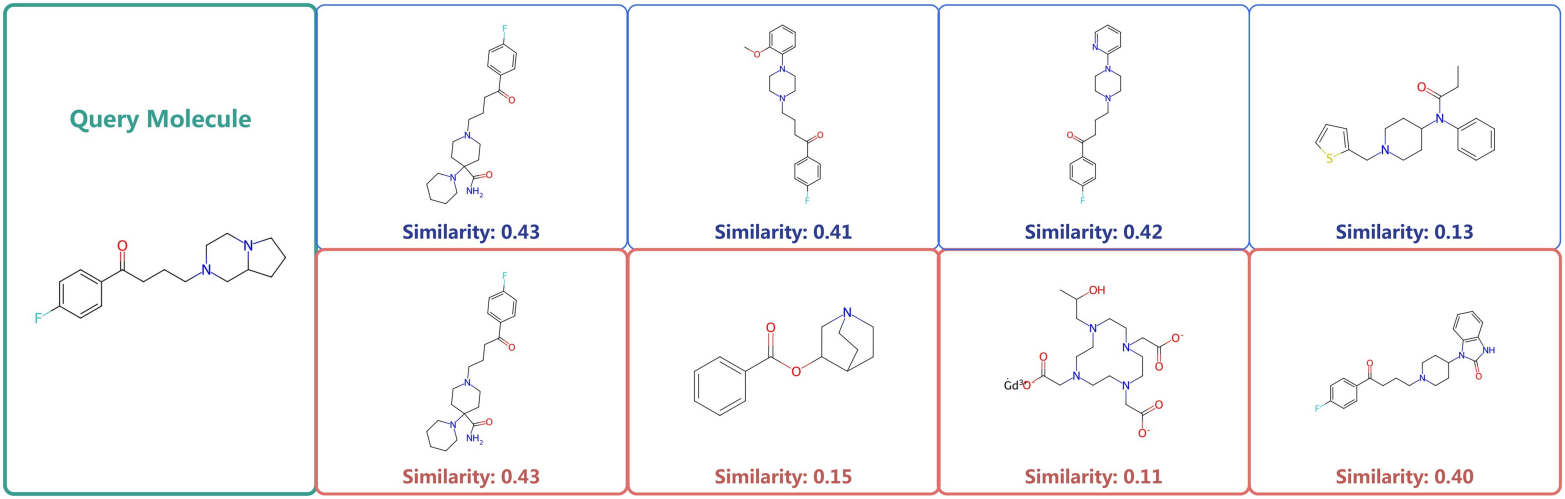
The image showcases the query molecule along with the four nearest molecules and their respective extracted representations. Tanimoto similarity scores, depicted beneath each molecule, quantify their chemical resemblance to the query molecule. The highlighted red sections denote the results from MoleculeSDE, while the blue sections represent the results from GTAM

We also conducted molecular retrieval experiments to show the model’s ability to obtain representations with chemical significance for practical applications. We conducted the case study on the Toxcast dataset. In this experiment, we calculate the cosine similarities between the 256-dimensional embeddings of all molecules in the test set and a designated query molecule. Each molecule’s output embedding is represented as a 256-dimensional vector. Additionally, we assessed the Tanimoto similarity among the extended-connectivity fingerprints (ECFPs) of the query molecule and those in the test set. Figure 2 shows the top four molecules identified by our model and MoleculeSDE based on cosine similarity to the query molecule. This observation suggests that GTAM’s molecular representations exhibit a higher correlation with molecular fingerprints, demonstrating the model’s ability to capture and reflect the intrinsic chemical characteristics of molecules effectively. It shows that the molecular representations obtained by GTAM have a high consistency with the molecular fingerprint. The results confirm the effectiveness of our model in practical applications, as well as its superior molecular feature extraction capabilities.

## 4 Conclusions

In our study, we introduce GTAM, an innovative contrastive learning approach for molecular representation learning. This method not only can capture complex edge relationships and the multifaceted nature of molecular structures using a novel molecular encoder but also implements two designed loss functions to enhance joint learning. Through extensive experiments on eight molecular property prediction tasks and 20 molecular conformation tasks, GTAM has achieved state-of-the-art results in several of these. This demonstrates that GTAM is capable of efficiently extracting molecular feature information and integrating data across 2D graphs and 3D conformations modalities.

While these results are encouraging, there are still challenges to address. We will expand our pre-trained model’s application to a wider array of scenarios, such as predicting drug-drug and drug-target interactions. This expansion aims to further demonstrate the model’s versatility and potential impact in the field of pharmaceutical research.

### 4.1 Key points

- GTAM has achieved state-of-the-art results in both 2D and 3D molecular representation tasks, outperforming existing methods in 22 out of 28 tasks.
- GTAM introduces a new contrastive learning framework for molecular representation, incorporating innovative molecular encoders and a unique geometric triangle awareness mechanism.
- GTAM establishes two new training objectives that integrate edge information supervision during pretraining. This facilitates effective cross-modal information transfer and significantly boosts the model’s expressive capabilities.

## Supplementary Material

btae524_Supplementary_Data

## Supplementary data

Comprehensive implementation details, datasets, and additional experiments can be found in Supplementary Appendices A–C.
