# Supplementary material for: GTAM: a molecular pretraining model with geometric triangle awareness
Source: Bioinformatics. 2024 Aug 23;40(9):btae524. doi: 10.1093/bioinformatics/btae524 (PMC11405089; doi:10.1093/bioinformatics/btae524)
Supplement: btae524_Supplementary_Data [file btae524_supplementary_data.pdf]

Supplementary Materials for GTAM: A Molecular Pretraining Model with Geometric Triangle Awareness

A Implementation Details

In this section, we illustrate the details of our proposed GTAM, including the featurization, time complexity, hyperparameters, etc.

A.1 Molecule Featurization

The molecule featurization is an essential factor that should be taken into consideration. For 2D graphs, atom, and edge featurization are considered in our models [Wu et al., 2018, Hu et al., 2020]. In the case of 3D conformations, we utilize solely the atom type information. More details can be found in Table S1.

Table S1: Featurization for atoms and edges.

	Hyperparameter	Value	2D Graph	3D Conformation
Atom Feature	Atom Type	[0, 118]	✓	✓
	Atomic Coordinate	$\mathbb{R}^3$	-	✓
	Atom Chirality	{ UNSPECIFIED, TETRAHEDRAL_CW TETRAHEDRAL_CCW, OTHER, misc }	✓	-
	Atom Degree	{ 0, 1, 2, 3, 4, 5, 6, 7, 8, 9, 10, misc }	✓	-
	Formal Charge	{ -5, -4, -3, -2, -1, 0, 1, 2, 3, 4, 5, misc }	✓	-
	Number H	{ 0, 1, 2, 3, 4, 5, 6, 7, 8, misc }	✓	-
	Number Radical E	{ 0, 1, 2, 3, 4, misc }	✓	-
	Hybridization	{ SP, SP2, SP3, SP3D, SP3D2, misc }	✓	-
	Is Aromatic	{ False, True }	✓	-
	Is in Ring	{ False, True }	✓	-
Edge feature	Edge Type	{ SINGLE, DOUBLE, TRIPLE, AROMATIC, misc }	✓	-
	Edge StereoType	{ STEREONONE, STEREOZ, STEREOE, STEREOCIS, STEREOTRANS, STEREOANY }	✓	-
	Is Conjugated	{ False, True }	✓	-
	Distance Map	$\mathbb{R}$	-	✓

A.2 Time complexity of geometric triangle awareness update mechanism

In analyzing the time complexity of the geometric triangle awareness update mechanism within the context of a graph characterized by n nodes and m edges, it's essential to break

down the complexity across the different components of the update mechanism—namely, node-to-edge, edge-to-node, and edge-to-edge components.

The transformation involved in the node-to-edge components primarily consists of linear projections. Given this operation's linear nature with respect to the number of nodes, the time complexity for the node-to-edge update process is $O(n)$. This efficiency stems from the direct correlation between the number of nodes and the computational steps required. Similarly, the edge-to-node components involve operations that are directly proportional to the number of edges, also resulting in a linear time complexity, specifically $O(m)$, under the assumption that each edge contributes independently to the computational load.

The core of the geometric triangle awareness update lies in the edge-to-edge component, which employs a self-attention mechanism to dynamically update edge embeddings based on their relationships with adjacent edges. Given the formulation:

$$a_{uvw} = \frac{\exp(\frac{1}{\sqrt{c}}\mathbf{q}^e_{uv}\mathbf{k}^e_{wv} + b^e_{wu})}{\sum\limits_{w \in \mathcal{N}(u)\cup\mathcal{N}(v)} \exp(\frac{1}{\sqrt{c}}\mathbf{q}^e_{uv}\mathbf{k}^e_{wv} + b^e_{wu})}, \tag{1}$$

$$z_{uv} = z_{uv} + \sigma(z_{uv}) \odot \Big(\sum_{w \in \mathcal{N}(u)\cup\mathcal{N}(v)} a_{uvw}\mathbf{v}^e_{uw} \Big). \tag{2}$$

where a_{uvw} is the attention weight for the edge (u, v) and w. The computation of the attention weight a_{uvw} requires consideration of all edges (u, v) and nodes w, thereby aggregating to a time complexity of $O(nm)$ for the entire graph. For fully connected graphs, the time complexity of our method is $O(n^3)$, as the number of edges is $O(n^2)$.

Therefore, the overall time complexity of the geometric triangle awareness update mechanism within GTAM is governed by the edge-to-edge component, summing up to $O(nm)$ for general graphs.

A.3 Implementation of GTAInfomax

GTAInfomax seeks to maximize the mutual information (MI) between latent 2D node representations h^{2D} from GTA-2D and 3D node representations h^{3D} derived from GTA-3D. The underlying intention is to amplify the concordance between h^{2D} and h^{3D} when they originate from the same molecule. The MI Equation can be formulated as:

$$\begin{aligned}\mathcal{L}_{\text{MI}} = -\frac{1}{2}\mathbb{E}_i\Big[&\log \frac{\exp(f_{h^{2D}}(h_i^{2D}, h_i^{3D}))}{\exp(f_{h^{2D}}(h_i^{2D}, h_i^{3D})) + \sum_{j\neq i}\exp(f_{h^{3D}}(h_j^{2D}, h_i^{3D}))} \\ &+ \log \frac{\exp(f_{h^{3D}}(h_i^{3D}, h_i^{2D}))}{\exp(f_{h^{3D}}(h_i^{3D}, h_i^{2D})) + \sum_{j\neq i}\exp(f_{h^{2D}}(h_j^{3D}, h_i^{2D}))}\Big],\end{aligned}$$

where $f_{h^{2D}}(h_i^{2D}, h_i^{3D})$, $f_{h^{3D}}(h_i^{3D}, h_i^{2D})$ are scoring functions for the two corresponding views, whose formulation can be quite flexible. Here we use $f_{h^{2D}}(h_i^{2D}, h_i^{3D}) = f_{h^{3D}}(h_i^{3D}, h_i^{2D}) = sim(h_i^{2D}, h_i^{3D})/\tau$.

We consider a set of N molecular graphs $\{\mathcal{G}_i^{2D}\}_{i=1}^N$ alongside their atomic coordinates $\{\mathbf{X}_i\}_{i=1}^N$. From these, our network generates multiple representations h_i^{2D} and h_i^{3D}. The contrastive learning methodology is driven by two primary goals: (1) Enhance the similarity of representations that form a positive pair h_i^{2D} and h_i^{3D}, particularly those derived from the same molecule, as denoted by the same index i. (2) Enhance the dissimilarity between negative pairs h_i^{2D} and h_j^{3D} where $i \neq j$, guaranteeing distinctiveness in 2D and 3D representations for disparate molecules.

Enhance the dissimilarity between negative pairs h_i^{2D} and where, fundamentally guaranteeing distinctiveness in 2D and 3D representations for disparate molecules.

Training objectives In GTAInfomax, the training objectives comprise our proposed novel objectives alongside the NTXent loss.

$$\mathcal{L} = \beta_1 \mathcal{L}_{\text{NTXent}} + \beta_2 \mathcal{L}_{\text{3Dedge}} + \beta_3 \mathcal{L}_{\text{2Dedge}}, \tag{3}$$

$$\mathcal{L}_{\text{NTXent}} = -\frac{1}{N}\sum_{i=1}^{N}\Big[\log \frac{\exp(sim(h_i^{2D}, h_i^{3D})/\tau)}{\sum_{j=1, i\neq j}^{N} \exp(sim(h_i^{2D}, h_j^{3D})/\tau)}\Big],$$

where $sim(h_i^{2D}, h_i^{3D}) = h_i^{2D} \cdot h_i^{3D} / (\|h_i^{2D}\| \|h_i^{3D}\|)$ is the cosine similarity and τ is a temperature parameter which can be seen as the weight for the most similar negative pair.

A.4 Parameter Details

Pretraining The GTAM model is pre-trained for 50 epochs using Adam optimizer with an initial learning rate of 1×10^{-4}, batch size 3096, and is trained on one A100 GPU. The parameters α_1, α_2, and α_3 are all set to 1. The parameters α_4 and α_5 are all set to 0.1.

Similar to the GTAM model, GTAInfomax also undergoes pretraining on the PCQM4Mv2 dataset. The GTAInfomax employs the Adam optimizer with an initial learning rate of 8×10^{-5} and a batch size of 2048. The parameter β_1 is set to 1. The parameters β_2 and β_3 are all set to 0.1. During pretraining, the learning rate schedule commences with 1500 optimization steps involving linear warmup, succeeded by the schedule dictated by the ReduceLROnPlateau scheduler. This scheduler utilizes a reduction factor of 0.5, a patience setting of 25 epochs, and a cooldown period of 20 epochs.

Finetuning 2D Graph Tasks For the molecular property prediction tasks with 2D information only, we use Adam with a start learning rate of 1×10^{-3} and a batch size of 32. For these datasets, we progressively escalate the learning rate from 0 to the initial learning rate using linear interpolation. Subsequently, for GTAInfomax, we employ the learning rate schedule as defined by ReduceLROnPlateau, incorporating a reduction factor of 0.5, a patience interval of 25 epochs, and a cooldown period of 20 epochs.

Finetunig 3D Conformation Tasks For the 3D conformation tasks, we use Adam with a start learning rate of 1×10^{-4} and a batch size of 128. The learning rate schedule employed is identical to that used for the 2D graph tasks.

Table S2: Hyperparameter specifications for GTAM

Hyperparameter	Value	Chosen Value
epochs	{50, 100}	50
Hidden dimension	{128, 256, 512}	256
Dropout	{0, 0.1, 0.2, 0.3}	0.3
pretraining learning rate	{1e-3, 1e-4, 1e-5}	1e-4
α_1	[0, 1]	1
α_2	[0, 1]	1
α_3	[0, 1]	1
α_4	[0, 1]	0.1
α_5	[0, 1]	0.1

Finetuning Broader Range of Tasks We use Adam with a start learning rate of 1×10^{-3} and a batch size of 32. Concurrently, we set the epoch values at 1 and 20. The learning rate schedule employed is identical to that used for the 2D graph tasks.

B Details of Dataset

B.1 Dataset

We use the PCQM4Mv2 dataset to pretrain and three datasets to finetune and evaluate our model.

The PCQM4Mv2 dataset, derived from the PubChemQC project, focuses on the machine learning task of predicting the HOMO-LUMO energy gap in molecules using their 2D graphs. This gap is a key quantum chemical property related to molecular reactivity, photoexcitation, and charge transport. Predicting this property from 2D graphs is advantageous as it avoids the costly process of obtaining 3D equilibrium structures through DFT-based geometry optimization.

The dataset presents molecules in the form of SMILES strings, which can be converted into 2D graphs. In these graphs, each node is characterized by a 9-dimensional feature vector, encompassing aspects like atomic number and chirality. Similarly, each edge is represented by a 3-dimensional feature vector, detailing elements such as bond type and stereochemistry.

We finetune our model using diverse datasets spanning various fields such as quantum mechanics, physical chemistry, biophysics, and physiology. This helps assess the effectiveness of our 3D pretraining across these domains. Specifically, for quantum mechanical properties, which depend on 3D geometric conformations, the incorporation of 3D information has proven highly beneficial, as indicated by several studies. Our focus is on evaluating how effectively our method can utilize this 3D information and apply it to molecules lacking

Table S3: Statistics of the used datasets.

Dataset	Compounds	Avg. Atoms	Avg. Bonds	SPLIT	Task Type	2D Graph	3D Conformation
PCQM4Mv2	3,378,606	14.2	29.2	-	-	✓	✓
BACE	1,513	34.1	36.9	scaffold	Classification	✓	-
BBBP	2,039	24.1	26.0	scaffold	Classification	✓	-
ClinTox	1,478	26.2	27.9	scaffold	Classification	✓	-
HIV	41,127	25.5	27.5	scaffold	Classification	✓	-
Sider	1,427	33.6	35.4	scaffold	Classification	✓	-
Tox21	7,831	18.6	19.3	scaffold	Classification	✓	-
ToxCast	8,575	18.8	19.3	scaffold	Classification	✓	-
MUV	93,087	24.2	52.6	scaffold	Classification	✓	-
ESOL	1,128	13.3	27.1	scaffold	Regression	✓	-
FreeSolv	642	8.7	16.8	scaffold	Regression	✓	-
Lipophilicity	4,200	27.0	59.0	scaffold	Regression	✓	-
QM9	133,385	18.0	18.6	random	Regression	✓	✓
MD17	442,790	-	-	random	Regression	-	✓

3D geometric information.

B.2 Units and Meaning of Quantum Properties

Table S4 displays the units for the QM9 dataset, with all results shown in Table 2 in main paper uniformly presented in the unit of milli-electron volts (meV).

Table S4: Units and description of quantum mechanical properties of the QM9 dataset.

Property	Unit	Description
Alpha	$Bohr^3$	Isotropic polarizability
Gap	eV	Gap between HOMO and LUMO
Homo	eV	Highest occupied molecular orbital energy
Lumo	eV	Lowest unoccupied molecular orbital energy
Mu	Debyte	Dipole moment
Cv	$\frac{cal}{molK}$	Heat capavity at 298.15K
G298	eV	Free energy at 298.15K
H298	eV	Enthalpy at 298.15K
R2	$Bohr^2$	Electronic spatial extent
U298	eV	Internal energy at 298.15K
U0	eV	Internal energy at 0K
Zpve	meV	Zero point vibrational energy

Table S5: Results on 3 regression tasks with 2D topological graphs only. The evaluation is Root Mean Square Error (RMSE). The performance at epoch 1 is represented on the left, while the performance after convergence is shown on the right. The best results are marked **bold**.

Model	3DInfomax	MoleculeSDE	GTAM
ESOL	2.95 / 1.19	4.02 / 0.98	**1.19** / **0.82**
FreeSolv	5.58 / 2.83	6.67 / 2.67	**3.18** / **1.83**
Lipophilicity	2.24 / 0.77	1.85 / 0.72	**0.83** / **0.68**

C Additional Experiments

C.1 Additional Experiment Results

To further demonstrate the capabilities of our model in multi-modal information integration and transferability, we considered 3 extra regression property prediction tasks that are strongly related to 3D structures [Crum-Brown and Fraser, 1865, Hansch and Fujita, 1964] using 2D graph. We selected two representative and top-performing multi-modal contrastive learning works, 3D Infomax and MoleculeSDE, for evaluation. As evident from the data in Table S5, GTAM exhibits outstanding performance even at epoch=1. It demonstrates that GTAM effectively transfers knowledge during the pretraining phase across different molecular representations, showing robust transferability in subsequent tasks. Furthermore, GTAM achieves exemplary results after convergence. These results indicate that GTAM, utilizing our designed loss functions, effectively integrates knowledge of 3D conformations into the 2D graph embeddings. Such integration enhances the model's transferability in tasks closely associated with 3D structural properties.

C.2 Ablation Studies

Aiming to further verify the effectiveness of GTAM, we conducted a series of ablation experiments, specifically:(1) Without two designed loss functions named GTAM (w/o loss). (2) Without the GTA-2D named GTAM (w/o 2D). (3) Without the GTA-3D named GTAM (w/o 3D). (4) applying our designed loss function used in the base MoleculeSDE model named GTAM(loss). (5) Replace the edge-to-edge update of geometric triangle awareness update mechanism in GTA-2D and GTA-3D with MLP named GTAM (MLP). The ablation results are included in Table S6 and Table S7.

Firstly, the results of GTA (w/o loss) in Table S6 and Table S7 illustrate that our designed loss functions significantly improve performance in both 2D graph and 3D conformation tasks. This suggests that our designed loss functions can more effectively facilitate more meaningful edge embedding across different molecular representations by adding direct edge constraints. On the MoleculeNet and QM9 datasets, GTAM compared with

Table S6: Ablation studies on MoleculeNet dataset with 2D topological graphs only. For each task, we present the mean ROC-AUC (with standard deviation) across three seeds, using scaffold splitting. The best results are marked **bold**.

Dataset	BACE	BBBP	ClinTox	HIV	Sider	Tox21	ToxCast	MUV	Avg
GTAM (w/o loss)	**85.31±0.44**	**71.13±1.04**	87.43±0.29	77.18±0.36	63.22±1.11	76.46±0.83	65.76±0.32	77.26±1.31	75.47±0.71
GTAM (w/o 3D)	84.12±0.33	69.97±0.77	86.13±0.82	**78.40±0.51**	62.59±0.75	76.63±1.17	65.62±0.39	76.01±1.43	74.93±0.77
GTAM (w/o 2D)	82.92±0.37	69.72±2.54	84.69±1.95	77.07±0.94	59.48±0.78	75.56±0.38	63.41±0.25	78.25±2.14	73.89±1.17
GTAM (loss)	80.89±0.29	68.67±2.74	85.57±2.03	77.00±0.77	60.11±0.81	76.98±0.34	65.97±0.31	79.76±2.01	74.37±1.16
GTAM (MLP)	83.02±0.27	69.95±0.71	86.77±0.91	76.83±0.47	62.42±0.83	77.09±0.64	64.84±0.19	77.81±1.74	74.84±0.72
GTAM	85.23±0.16	70.65±0.49	**88.37±0.53**	77.52±0.22	**63.89±0.62**	**77.59±0.36**	**66.48±0.14**	**79.51±0.47**	**76.13±0.37**

Table S7: Ablation studies on MD17 dataset with 3D geometric conformations only. The evaluation is Mean Absolute Error (MAE). The best results are marked **bold**.

Dataset	Aspirin	Benzene	Ethanol	Malonaldehyde	Naphthalene	Salicylic	Toluene	Uracil
GTAM (w/o loss)	0.8451	0.2716	0.2866	0.5079	0.3494	0.7048	0.4113	0.4797
GTAM (w/o 3D)	1.0582	0.2703	0.2988	0.5197	0.3763	0.7071	0.4989	0.4618
GTAM (w/o 2D)	0.8443	0.2671	0.2845	0.5127	0.3511	0.7036	0.4196	0.4850
GTAM (loss)	0.9886	0.2895	0.2942	0.5083	0.4139	0.7091	0.4771	0.4493
GTAM (MLP)	0.9471	0.2899	0.2889	0.5048	0.3812	0.7002	0.4516	0.4564
GTAM	**0.8437**	**0.2658**	**0.2822**	**0.5008**	**0.3442**	**0.6981**	**0.4063**	**0.4552**

GTAM (MLP) shows significant improvements after adding the geometric triangle awareness update to the edge-to-edge update method in almost all tasks. It demonstrates the superiority of our update approach.

Secondly, in the molecular property predictions, the GTAM (w/o 2D) achieved poor results compared to the final model, which demonstrates that GTA-2D enhances the aggregation of 2D information effectively by adding edge-to-edge updates. The accuracy of the GTAM (w/o 3D) was lower than that of our final model, which shows that the use of GTA-3D plays a significant role in the integration of 3D information into 2D information.

Finally, in the MD17 force predictions, we also test the GTAM (w/o 3D) and the GTAM (w/o 2D). Their performance on the dataset was lower than that of the final model, highlighting the information aggregation capabilities of both the GTA-2D and GTA-3D. Furthermore, the utilization of a uniform information aggregation approach across both modalities aids in the synergistic learning of multi-modal data.

References

A Crum-Brown and Thomas R Fraser. On the Connection between Chemical Constitution and Physiological Action. *Trans R Soc Edinb*, 25(1968-1969):257, 1865.

Corwin Hansch and Toshio Fujita. p-σ-π Analysis. A Method for the Correlation of Biological Activity and Chemical Structure. *Journal of the American Chemical Society*, 86 (8):1616–1626, 1964.

Weihua Hu, Matthias Fey, Marinka Zitnik, Yuxiao Dong, Hongyu Ren, Bowen Liu, Michele

Catasta, and Jure Leskovec. Open Graph Benchmark: Datasets for Machine Learning on Graphs. *Advances in neural information processing systems*, 33:22118–22133, 2020.

Zhenqin Wu, Bharath Ramsundar, Evan N Feinberg, Joseph Gomes, Caleb Geniesse, Aneesh S Pappu, Karl Leswing, and Vijay Pande. MoleculeNet: A Benchmark for Molecular Machine Learning. *Chemical science*, 9(2):513–530, 2018.
